# Automated dose-gradient curve and dose-volume histogram analysis platform: development, validation, and clinical decision support with TG-119 datasets

**DOI:** 10.3389/fonc.2026.1826856

**Published:** 2026-05-20

**Authors:** Han-Back Shin, Wonyoung Cho, Young Eun Choi, Jin Sung Kim, KiHoon Sung

**Affiliations:** 1 Department of Radiation Oncology, Gachon University Gil Medical Center, Incheon, Republic of Korea; 2 Oncosoft Inc., Seoul, Republic of Korea; 3 Department of Radiation Oncology, Yonsei Cancer Center, Heavy Ion Therapy Research Institute, Yonsei University College of Medicine, Seoul, Republic of Korea; 4 Department of Radiation Oncology, Gachon University College of Medicine, Incheon, Republic of Korea

**Keywords:** dose fall-off, dose gradient curve, dose-volume histogram, plan quality evaluation, stereotactic radiotherapy

## Abstract

**Background:**

Highly conformal radiotherapy techniques, such as stereotactic radiosurgery and stereotactic ablative radiotherapy, require steep dose fall-off to spare organs at risk (OARs). The dose-volume histogram (DVH) provides limited spatial information, whereas the dose gradient curve (DGC) offers quantitative assessment of dose fall-off but has seen limited clinical use due to time-consuming manual processing.

**Purpose:**

This study aimed to develop and validate an automated DGC and DVH analysis platform to enable accurate and rapid dose gradient evaluation for radiotherapy plan assessment.

**Methods:**

The platform automatically processes DICOM RT dose and RT structure files, with optional RT Plan input for direct prescription dose extraction, to compute differential and cumulative dose gradient index (dDGI and cDGI), DVH metrics, and clinically informed dose gradient quality grading. Computational validation was performed using AAPM TG-119 phantom datasets, including prostate, head and neck, C-shape, and multi-target cases, at 1.25 mm and 2.5 mm dose grid resolutions. Clinical validation was conducted using 142 Gamma Knife stereotactic radiosurgery plans for vestibular schwannoma from the publicly available Vestibular-Schwannoma-SEG dataset (The Cancer Imaging Archive).

**Results:**

The platform reduced computation time from over 2 hours (original R-based workflow) to under 10 seconds, representing >99.8% efficiency improvement. All TG-119 cases met or exceeded recommended PTV coverage and OAR tolerances. Using prescription doses extracted from DICOM RT Plan file and marching-cubes-based surface area estimation, dDGI at the prescription dose ranged from 0.312 to 0.792 mm on the 1.25 mm grid and from 0.327 to 0.659 mm on the 2.5 mm grid; cDGI at 50% of the prescription dose ranged from 12.4 to 15.7 mm (1.25 mm) and from 11.7 to 15.5 mm (2.5 mm). Clinical validation with 142 vestibular schwannoma Gamma Knife SRS cases (median target volume 0.881 cc, range 0.036–7.183 cc; Rx = 12–13 Gy) yielded dDGI at Rx = 0.294±0.089 mm with Paddick conformity index = 0.584±0.075 and gradient index = 2.770±0.218. The dDGI was strongly correlated with target volume (Pearson r=0.885, $p\;<\;10^{-48}$) but independent of conformity index ( r=0.040, p = 0.634), confirming that gradient steepness and conformity assess distinct plan quality aspects. Paired T1–T2 MRI-based reproducibility analysis ( n=30) demonstrated excellent agreement (dDGI r=0.987, mean difference 3.3%).

**Conclusions:**

The proposed platform enables rapid, automated DGC analysis practical for routine plan evaluation. The integrated quality grading bridges the gap between quantitative dose gradient metrics and actionable clinical decisions, complementing conventional DVH-based assessment.

## Introduction

Highly conformal radiotherapy techniques, such as stereotactic radiosurgery (SRS) and stereotactic ablative radiotherapy (SABR), require steep dose fall-off outside the target volume to minimize irradiation of adjacent normal tissues (1, 2). Quantitative evaluation of the dose gradient is therefore essential for treatment plan assessment and comparison.

The dose-volume histogram (DVH) remains the standard tool for plan evaluation (3); however, it provides limited information about the spatial distribution of dose outside the target (4, 5). DVH curves of competing plans frequently appear nearly identical near the prescription dose, even when their dose fall-off characteristics differ substantially (5). Conventional indices such as the conformity index (CI) (6, 7), gradient index (GI) (5, 8), and volume-ratio metrics (e.g., R50%) (9) each address specific aspects of the dose distribution but do not capture the full profile of dose fall-off, and their reliability depends on target volume and shape (10).

To address these limitations, the dose gradient curve (DGC) was introduced as a distance-based metric that evaluates dose fall-off by analyzing concentric isodose shells surrounding the target volume (4). The DGC provides differential and cumulative dose gradient indices (dDGI and cDGI), offering an intuitive and quantitative assessment of how rapidly dose decreases with increasing distance from the target.

Despite its clinical utility, the adoption of DGC analysis has been limited by practical barriers. The original workflow required exporting isodose structure coordinates via third-party software (Mirada RTx) and processing them in the R statistical environment (4); in our experience, this procedure took over 2 hours per plan. Furthermore, the analysis output lacked clinical interpretation guidelines, requiring expert knowledge to translate DGC metrics into actionable plan quality assessments.

This study presents an automated web-based platform that addresses these limitations through two key innovations: (1) a computational pipeline reducing analysis time from hours to seconds, and (2) integrated clinical decision support providing clinically informed quality grading of dose gradient metrics. The platform was validated using AAPM TG-119 phantom datasets (11).

## Materials and methods

### System architecture

**Figure 1:**
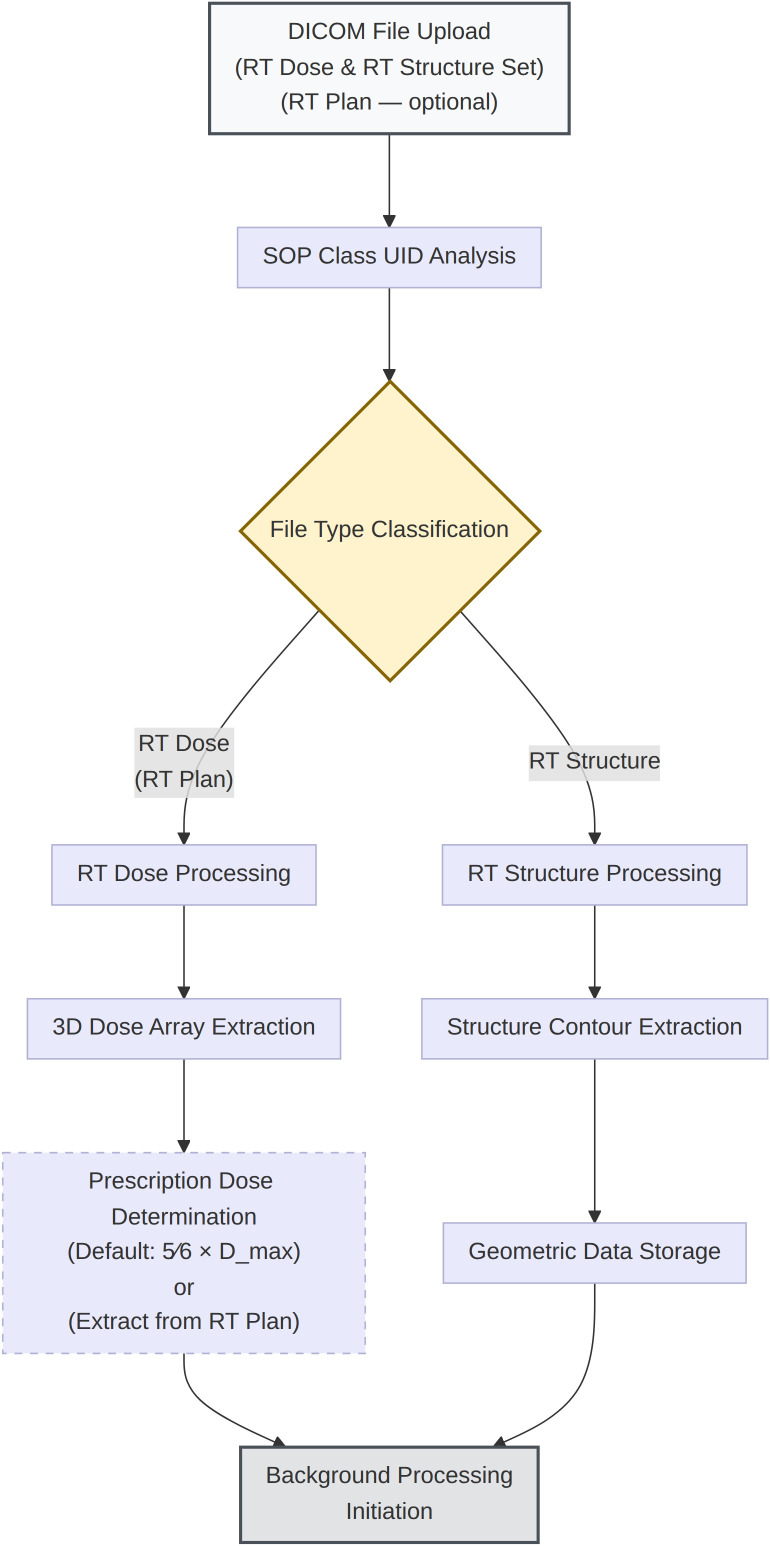
Uploaded DICOM files undergo SOP Class UID analysis to classify RT Dose, RT Structure Set, and (optional) RT Plan objects. RT Dose files are parsed to extract the three-dimensional dose array. When an RT Plan file is provided, the prescription dose is extracted directly from the DICOM metadata; otherwise, the system estimates a default value 5/6 × Dmax (user-adjustable). Dashed boxes indicate optional or conditional processing paths. RT Structure Set files are processed to extract structure contour geometry. Both branches feed into the next computation stage.

**Figure 2:**
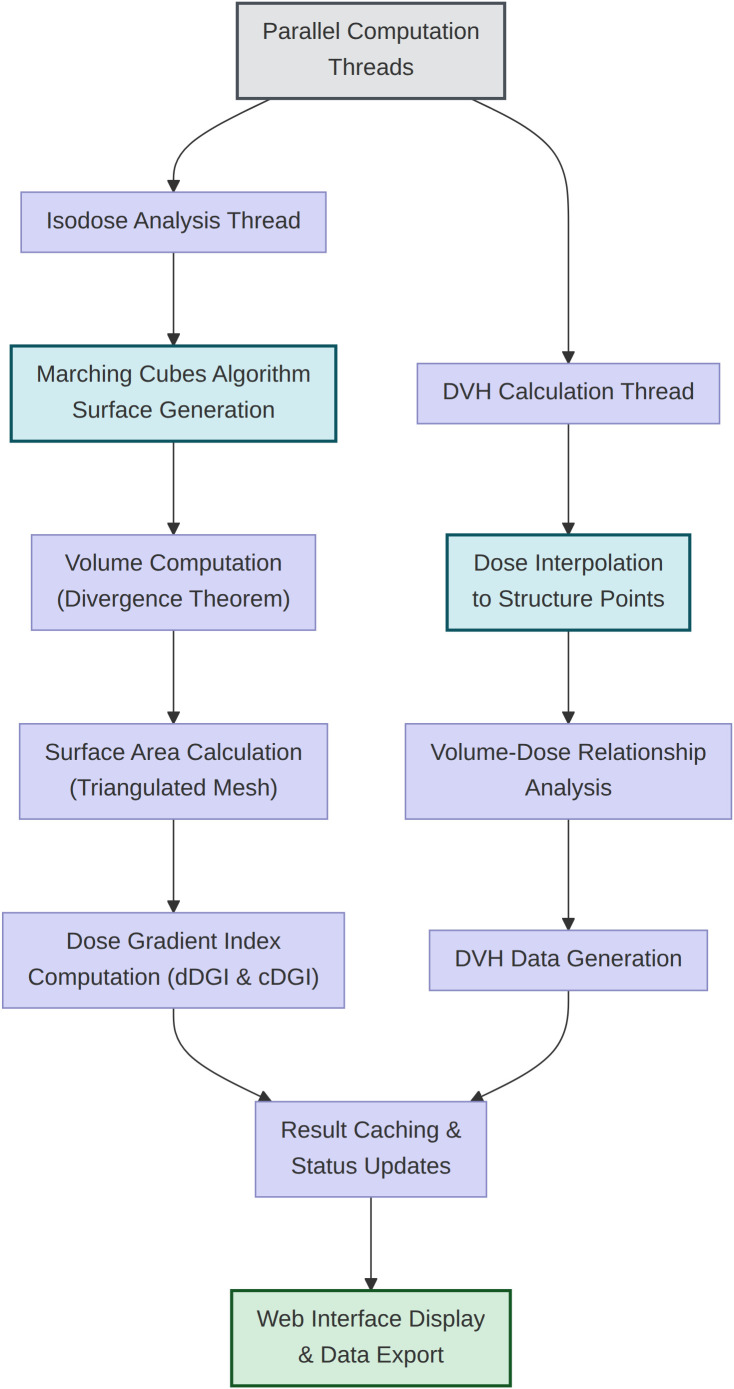
Two parallel threads process the ingested data. The isodose analysis thread generates triangulated isosurfaces via the marching cubes algorithm, computes enclosed volumes and surface areas, and derives differential and cumulative dose gradient indices. The DVH calculation thread interpolates dose values to structure contour points and generates dose-volume histogram data. Results are cached and delivered to the web interface.

The platform employs a client–server architecture consisting of a Python-based backend and a responsive HTML5/JavaScript frontend (**Figures 1**, **2**). The backend was implemented in Python 3.12 using Flask 2.2 as the web application framework. Key computational dependencies include pydicom 3.0 for DICOM file parsing and manipulation, NumPy 1.26 for numerical array operations, scikit-image 0.24 for three-dimensional image processing, pandas 2.2 for data manipulation, and dicompyler-core 0.5 for voxel-based DVH calculation.

The frontend interface was built using HTML5, CSS3, and JavaScript with Chart.js 4.4 for interactive data visualization. The system operates on a lightweight Flask server compatible with Web Server Gateway Interface (WSGI) deployment, and cross-origin resource sharing (CORS) enables distributed network access.

### DICOM data preprocessing

The system accepts RT Dose and RT Structure Set DICOM files through a browser-based upload interface with automated file type identification. File classification is performed using standardized DICOM SOP Class UIDs, eliminating manual file sorting.

Upon receipt, RT Dose files undergo extraction of the three-dimensional dose array from DICOM pixel data, conversion to physical dose units via the DoseGridScaling factor, and spatial calibration using PixelSpacing and GridFrameOffsetVector parameters. When an RT Plan file is uploaded alongside the dose and structure files, the prescription dose and fractionation schedule are automatically extracted from the ‘DoseReferenceSequence’ and ‘FractionGroupSequence’; in the absence of an RT Plan file, the system automatically estimates a default prescription dose as 5/6 of the maximum dose value, corresponding to approximately the 83% isodose level commonly used in SRS/SABR dose prescription (12, 13). In either case, this value is user-adjustable to match the actual clinical prescription.

RT Structure Set files are processed to extract ROI contour coordinates from the Structure-SetROISequence. Contour integrity and spatial consistency with the dose grid are validated to ensure accurate interpolation during DVH generation. Patient demographic information (name, ID, sex, age) is extracted from DICOM headers and displayed in the interface for verification.

### Dose gradient index computation

The dDGI quantifies the average distance between adjacent isodose surfaces and is computed as (4):


$$dDGI_{d}=\frac{\left[V(d)-V(d+\text{Δ}d)\right]}{\frac{1}{2}\times [S(d)+S(d+\text{Δ}d)]}\;,$$

where *V(d)* and *S(d)* denote the volume and surface area enclosed by the isodose surface at dose level *d*, and Δ*d* is the user-configurable step (dose interval). Because dDGI scales proportionally with Δ*d*, values are normalized by the step size to enable comparison across different calculation settings (4):


$${\text{dDGI}}_{\text{N}}=\frac{\text{dDGI}}{\text{Δ}d}\;.$$

For each isodose level, the enclosed volume is computed as the product of the number of voxels at or above that dose threshold and the voxel volume derived from DICOM spatial parameters. The isodose surface area is computed by default using the marching cubes algorithm (14), which extracts a triangulated isosurface at the 0.5 level of the binary dose mask via scikit-image and sums the exact triangle areas from cross-products. An alternative voxel-based method using six-connectivity boundary detection is also available for faster but approximate estimation (**Supplementary Table 1**).

The cDGI represents the integrated distance from the reference dose surface to each isodose level:


$$cDGI_{i}=\;\sum_{j=i}^{D_{0}-\Delta d\;}dDGI_{j}\;,\;cDGI_{D_{0}}=0\;,$$

where *D_0_* denotes the reference dose (prescription dose by default). Dose levels are generated symmetrically around the prescription dose with the user-specified step size, ensuring that the prescription dose always appears as an explicit calculation point. Both Gy-based and percentage-based step sizes are supported, with automatic interconversion.

### DVH computation and metrics

DVH calculation employs the dicompyler-core library, which implements a voxel-based sampling algorithm. For each structure, the algorithm creates Boolean masks using point-in-polygon testing on each contour plane, samples dose values at voxel locations within structure boundaries, and bins dose distributions at 1 cGy resolution. Differential histograms are converted to cumulative DVH format.

From the cumulative DVH, the platform automatically computes the following clinical metrics for each structure: $D_{x}$% metrics (dose covering x% of volume: $D_{98}$, $D_{95}$, $D_{90}$, $D_{50}$, $D_{10}$, $D_{5}$, $D_{2}$) were computed by linear interpolation on the cumulative DVH. $V_{x}$ Gy metrics (volume receiving ≥ x Gy: $V_{5}$– $V_{50}$ Gy at 5 Gy intervals) were computed by interpolation on the dose axis. Summary statistics included mean dose (trapezoidal integration of the cumulative DVH), maximum dose ( $D_{2}$ surrogate), and minimum dose ( $D_{98}$ surrogate).

Structure volumes are independently computed from RT Structure Set contour coordinates using the shoelace formula for polygon areas on each contour plane, multiplied by the inter-slice thickness.

### Clinical decision support: dose gradient quality grading

The dDGI at the prescription dose is classified into four quality grades: Ideal (<3 mm) indicates dDGI values typical of small-volume SRS plans with steep fall-off; Good (3–5 mm) corresponds to values commonly observed in standard stereotactic body radiotherapy (SBRT) plans; Acceptable (5–7 mm) is consistent with larger targets or low-density tissue (e.g., lung); and Poor (>7 mm) suggests a shallow gradient for which plan re-optimization may be warranted. The thresholds were further informed by dDGI ranges from virtual-target SRS plans in the original DGC study (4) and the relationship between gradient steepness and target size (15). The Ideal threshold of 3 mm is grounded in the established SRS dosimetric benchmark (8), in which the optimum gradient score defined as a physical fall-off distance of 3 mm or less from the prescription isodose to the half-prescription isodose surface–a criterion subsequently referenced in widely adopted gradient evaluation frameworks (5). Clinical interpretation text is automatically generated based on the assigned grade, providing immediate feedback to facilitate plan review.

### Validation

Computational validation was performed using the AAPM TG-119 test suite (11), which includes prostate, head-and-neck, C-shape, and multi-target phantom configurations. For each case, treatment plans were generated using the Eclipse treatment planning system (TPS; Varian Medical Systems, Palo Alto, CA, USA) and exported at 1.25 and 2.5 mm dose grid resolutions. Planning target volume (PTV) dose coverage and OAR doses were compared against TG-119 goals, and computational efficiency was benchmarked against the original R-based DGC workflow (4).

Clinical validation was conducted using the Vestibular-Schwannoma-SEG dataset (16), a publicly available collection of 242 consecutive patients with vestibular schwannoma treated with Gamma Knife SRS at a single institution (The Cancer Imaging Archive). We analyzed 142 cases that included both the target (tumor volume, TV) and at least one OAR (cochlea), with matched RT Dose, RT Plan, and RT Structure triplets verified through DICOM reference chains. Prescription doses (Rx) were 13 Gy ( n=120) or 12 Gy ( n=22); dose grids were 1.0 mm isotropic. For each case, dDGI and cDGI were computed alongside Paddick CI (6), GI ( $V_{50\%Rx}/V_{Rx}$) (5), and $R_{50\%}$. Cochlea doses (maximum and mean) were extracted from DVH analysis.

### Statistical analysis

Statistical analyses were performed in Python 3.12 using SciPy 1.11 and pandas 2.2. Continuous variables are reported as mean ± standard deviation (SD); categorical counts are reported as n (%). Associations between DGC metrics and conventional dosimetric indices were assessed using Pearson ( r) and Spearman ( ρ) correlation coefficients with two-sided p-values, and interpreted using Cohen’s conventional thresholds (17). Exploratory subgroup analyses stratified by target volume and prescription dose are reported as descriptive summaries. Reproducibility across paired T1/T2-MR-based analyses was quantified using Pearson correlation and the mean absolute percentage difference, $mean_{i}(|x_{i}^{T1}-x_{i}^{T2}|/{\bar{x}}_{i}\times 100\%$. Given the exploratory nature of the analyses, p < 0.05 was regarded as significant without correction for multiple comparisons.

## Results

### Platform overview

**Figure 3:**
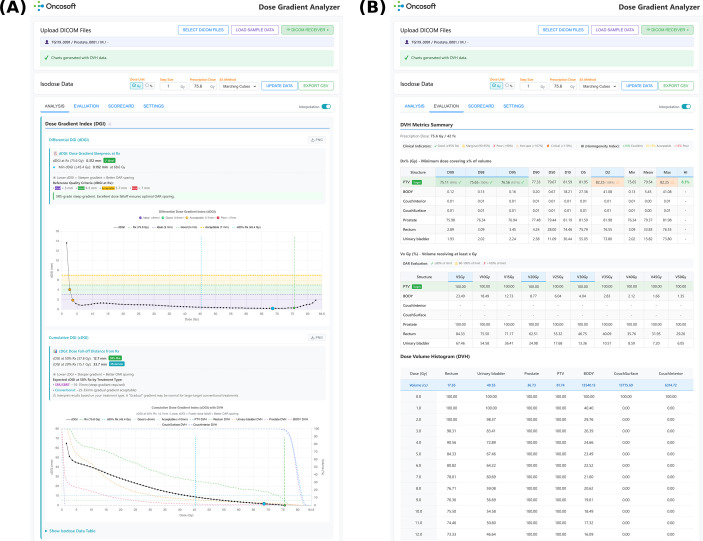
Platform interface has **(A)** analysis tab displaying differential and cumulative dose gradient index charts with clinical quality grading, reference quality criteria, and dose fall-off distance interpretation and **(B)** evaluation tab with structure-specific DVH metrics ( $D_{x}$%, $V_{x}$Gy) and clinical indicator assessment.

The developed platform (accessible at https://dgi.oncosoft.io, **Figure 3**) provides an integrated interface for DGC and DVH analysis. The interface comprises two primary functional tabs: (1) Analysis, displaying dDGI and cDGI charts with clinical quality grading and a tabular summary of dose, volume, surface area, dDGI, and cDGI values at each isodose level; and (2) Evaluation, presenting structure-specific DVH data, and metrics tables. The quantitative validation below focuses on the dDGI at the prescription dose as the primary metric because it directly reflects the dose fall-off in the clinically most relevant region; cDGI profiles were computed for all cases and are available through the interactive interface.

### Computational efficiency

The platform reduced DGC computation time from over 2 hours [measured using the original R-based workflow (4)] to less than 10 seconds, representing an efficiency improvement of >99.8%. The original workflow required three sequential manual steps: exporting isodose structure coordinates from the TPS via third-party software, loading and parsing those coordinates in R, and performing iterative mesh-based geometric calculations. The present platform eliminates the first two steps through direct DICOM parsing with pydicom and replaces the iterative calculations with vectorized NumPy array operations for isodose volume and surface area estimation.

### TG-119 phantom validation

The validation results across all four TG-119 phantom configurations are summarized in **Table 1**. PTV dose coverage met or exceeded TG-119 recommendations in all cases. OAR doses were within TG-119 tolerances: prostate rectum $D_{30}=4877$ cGy and bladder $D_{30}=1749$ cGy (goal <7000 cGy each); head-and-neck cord $D_{max}=3760$ cGy (goal <4000 cGy) and parotid $D_{50}=1486$ cGy (goal <2000 cGy); C-shape core $D_{10}=2051$ cGy (goal <2500 cGy). These OAR results confirm that the platform accurately reproduces dose metrics relevant to clinical plan acceptance.

**Table 1:** Validation results for the four TG-119 phantom configurations at 1.25 mm and 2.5 mm dose grid resolutions.

Case (Rx/fx)	Metric	TG-119 goal	1.25 mm grid	2.5 mm grid
Prostate (75.6 Gy/42 fx)	PTV $D_{95}$	>7560 cGy	7656 cGy	7700 cGy
	Rectum $D_{30}$	<7000 cGy	4876 cGy	–
	Bladder $D_{30}$	<7000 cGy	1748 cGy	–
	dDGI at Rx	–	0.312 mm	0.307 mm
	cDGI at 50% Rx	–	12.4 mm	11.7 mm
Head & Neck (50.0 Gy/25 fx)	PTV $D_{90}$	>5000 cGy	5049 cGy	5081 cGy
	PTV $D_{99}$	>4650 cGy	4952 cGy	4955 cGy
	Cord $D_{max}$	<4000 cGy	3760 cGy	–
	Parotid $D_{50}$	<2000 cGy	1485 cGy	–
	dDGI at Rx	–	0.702 mm	0.659 mm
	cDGI at 50% Rx	–	15.7 mm	15.8 mm
C-shape (50.0 Gy/25 fx)	PTV $D_{95}$	>5000 cGy	5089 cGy	5088 cGy
	Core $D_{10}$	<2500 cGy	2051 cGy	–
	dDGI at Rx	–	0.483 mm	0.485 mm
	cDGI at 50% Rx	–	13.4 mm	13.5 mm
Multi-target (50.0 Gy/25 fx)	Central $D_{99}$	>5000 cGy	5070 cGy	5087 cGy
	Superior $D_{99}$	>2500 cGy	2558 cGy	2578 cGy
	Inferior $D_{99}$	>1250 cGy	1318 cGy	1310 cGy
	dDGI at Rx	–	0.404 mm	0.362 mm
	cDGI at 50% Rx	–	12.4 mm	11.7 mm

Prescription doses (Rx) were extracted from DICOM RT Plan files and applied consistently to both grids. Organ at risk metrics are reported only for the 1.25 mm grid because DVH-based indices are largely insensitive to grid resolution; dashes (-) indicate metrics not separately tabulated.

cDGI, cumulative dose gradient index; dDGI, differential dose gradient index; $D_{x}$, dose received by x% of the structure volume; $D_{max}$, maximum point dose; fx, fraction; PTV, planning target volume; Rx, prescription dose.

Prescription doses were extracted from the DICOM RT Plan files accompanying each dataset and applied consistently to both dose grid resolutions (**Table 1**).

For the prostate phantom (Rx = 75.6 Gy/42 fx), PTV $D_{95}$ exceeded the TG-119 goal (>7560 cGy), achieving 7660 cGy (1.25 mm) and 7700 cGy (2.5 mm). Rectum and bladder doses were within acceptable limits. The dDGI at Rx was 0.312 mm (1.25 mm) and 0.327 mm (2.5 mm).

For the head-and-neck phantom (Rx = 50 Gy/42 fx), PTV $D_{90}$ and $D_{99}$ were consistent with TG-119 criteria. The dDGI at Rx was 0.702 mm (1.25 mm) and 0.659 mm (2.5 mm).

The C-shape phantom (Rx = 50 Gy/25 fx) achieved PTV $D_{95}$ >5000 cGy with acceptable core sparing. The dDGI at Rx was 0.483 mm (1.25 mm) and 0.485 mm (2.5 mm).

In the multi-target phantom (Rx = 50 Gy/25 fx), all targets satisfied TG-119 coverage and OAR constraints. The dDGI at Rx was 0.404 mm (1.25 mm) and 0.362 mm (2.5 mm).

Grid sensitivity of dDGI varied across cases. The prostate and C-shape cases showed the 1.25 mm grid yielding lower dDGI at Rx (steeper gradient) than the 2.5 mm grid, with differences of 4.9% and 0.4%, respectively. The C-shape case showed near-identical values across grids, consistent with its moderately concave geometry. The head-and-neck and multi-target case exhibited the opposite trend, with the 1.25 mm grid yielding higher dDGI (6.1% and 10.2%, respectively).

The cDGI at 50% of the prescription dose ranged from 12.4 to 15.7 mm across all cases on the 1.25 mm grid and from 11.7 to 15.5 mm on the 2.5 mm grid (**Table 1**). The head-and-neck case exhibited the largest cDGI at 50% Rx (15.7 mm on the 1.25 mm grid), whereas the prostate case showed the smallest (12.4 mm). In three of the four cases, the 1.25 mm grid produced higher cDGI values than the 2.5 mm grid; the C-shape case showed near-identical values across grid (13.4 vs. 13.5 mm).

### Clinical SRS validation

To evaluate the platform with clinical stereotactic data, 142 Gamma Knife SRS plans for vestibular schwannoma were analyzed from the publicly available Vestibular-Schwannoma-SEG dataset (16). The median target volume was 0.881 cc (range 0.036−7.183 cc), with prescription doses of 13 Gy ( n=120) or 12 Gy ( n=22) on 1.0 mm isotropic dose grids.

The dDGI at the prescription dose was 0.294±0.089 mm (median 0.277 mm, range 0.111–0.562 mm), with all values falling within the “Ideal” quality grade (<3 mm). The minimum dDGI was 0.287±0.093 mm. The cDGI at 50% Rx was 3.139±1.037 mm (range 1.371−5.952 mm), substantially lower than the TG-119 values ( 12.4−15.7 mm on the 1.25 mm grid), reflecting the compact dose distributions characteristic of single-fraction SRS.

Conventional dosimetric indices were computed for comparison: Paddick CI = 0.584±0.075 (range 0.403−0.903), GI = 2.770±0.218 (range 2.470−3.867), and $R_{50}=4.256\pm 0.601$ (range 2.767−6.444). Target dose coverage was adequate, with $D_{95}=12.67\pm 0.66$ Gy and $D_{99}=11.29\pm 1.04$ Gy. Cochlea doses were: maximum 5.12±2.05 Gy and mean 3.10±1.06 Gy. The complete results are summarized in **Table 2**.

**Table 2:** Clinical validation results from 142 vestibular schwannoma Gamma Knife SRS cases.

Metric	Mean ± SD	Median	Range
Target volume (cc)	1.467 ± 1.437	0.881	0.036–7.183
dDGI at Rx (mm)	0.294 ± 0.089	0.277	0.111–0.562
Min dDGI (mm)	0.287 ± 0.093	0.269	0.111–0.562
cDGI at 50% Rx (mm)	3.139 ± 1.037	2.971	1.371–5.952
Paddick CI	0.584 ± 0.075	0.579	0.403–0.903
GI	2.770 ± 0.218	2.731	2.470–3.867
$R_{50\%}$	4.256 ± 0.601	4.170	2.767–6.444
Target $D_{95}$ (Gy)	12.67 ± 0.66	12.89	8.43–13.86
Target $D_{99}$ (Gy)	11.29 ± 1.04	11.51	6.04–12.98
Cochlea max dose (Gy)	5.12 ± 2.05	4.38	1.72–12.74
Cochlea mean dose (Gy)	3.10 ± 1.06	2.83	1.11–6.78

All metrics were computed using the marching cubes surface area method on 1.0 mm isotropic dose grids. Target structures were labeled as tumor volume or acoustic neuroma, and the cochlea was the primary organ at risk evaluated.

CI, conformity index; cDGI, cumulative dose gradient index; dDGI, differential dose gradient index; $D_{x}$, dose received by x% of the structure volume; GI, gradient index; $,\;R_{50\%}$, ratio of 50% isodose volume to target volume; Rx, prescription dose; SD, standard deviation.

### Correlation and subgroup analysis

Pearson and Spearman correlation analyses were performed between DGC metrics and conventional dosimetric indices across the 142 clinical SRS cases (**Table 3**). The dDGI at Rx was strongly correlated with target volume (Pearson r=0.885, $p=2.6\times 10^{-48}$; Spearman ρ=0.922, $p=1.4\times 10^{-59}$), confirming that larger targets produce less steep dose fall-off. Notably, dDGI was statistically independent of Paddick CI (Pearson r=0.885, p=0.634), demonstrating that gradient steepness and target conformity assess distinct aspects of plan quality. The dDGI showed a weak negative correlation with GI ( r=−0.355, $p=1.5\times 10^{-5}$), while cDGI at 50% Rx exhibited a moderate negative correlation with GI ( r=−0.501, $p=2.1\times 10^{-10}$).

**Table 3:** Correlation analysis between DGC metrics and conventional dosimetric indices from the VS-SEG clinical dataset (n/142).

Metric pair	Pearson r	p-value	Spearman ρ	p-value
dDGI at Rx vs. TV	0.885	$2.6\times 10^{-48}$	0.922	$1.4\times 10^{-59}$
dDGI at Rx vs. GI	-0.355	$1.5\times 10^{-5}$	-0.371	$5.5\times 10^{-6}$
cDGI at 50% Rx vs. GI	-0.501	$2.1\times 10^{-10}$	-0.529	$1.3\times 10^{-11}$
dDGI at Rx vs. Paddick CI	0.040	0.634	0.310	$1.7\times 10^{-4}$
GI vs. TV	-0.447	$2.5\times 10^{-8}$	-0.614	$4.4\times 10^{-16}$

CI, conformity index; cDGI, cumulative dose gradient index; dDGI, differential dose gradient index; GI, gradient index; r, Pearson product-moment correlation coefficient; Rx, prescription dose; TV, target volume; ρ, Spearman rank correlation coefficient.

**Figure 4:**
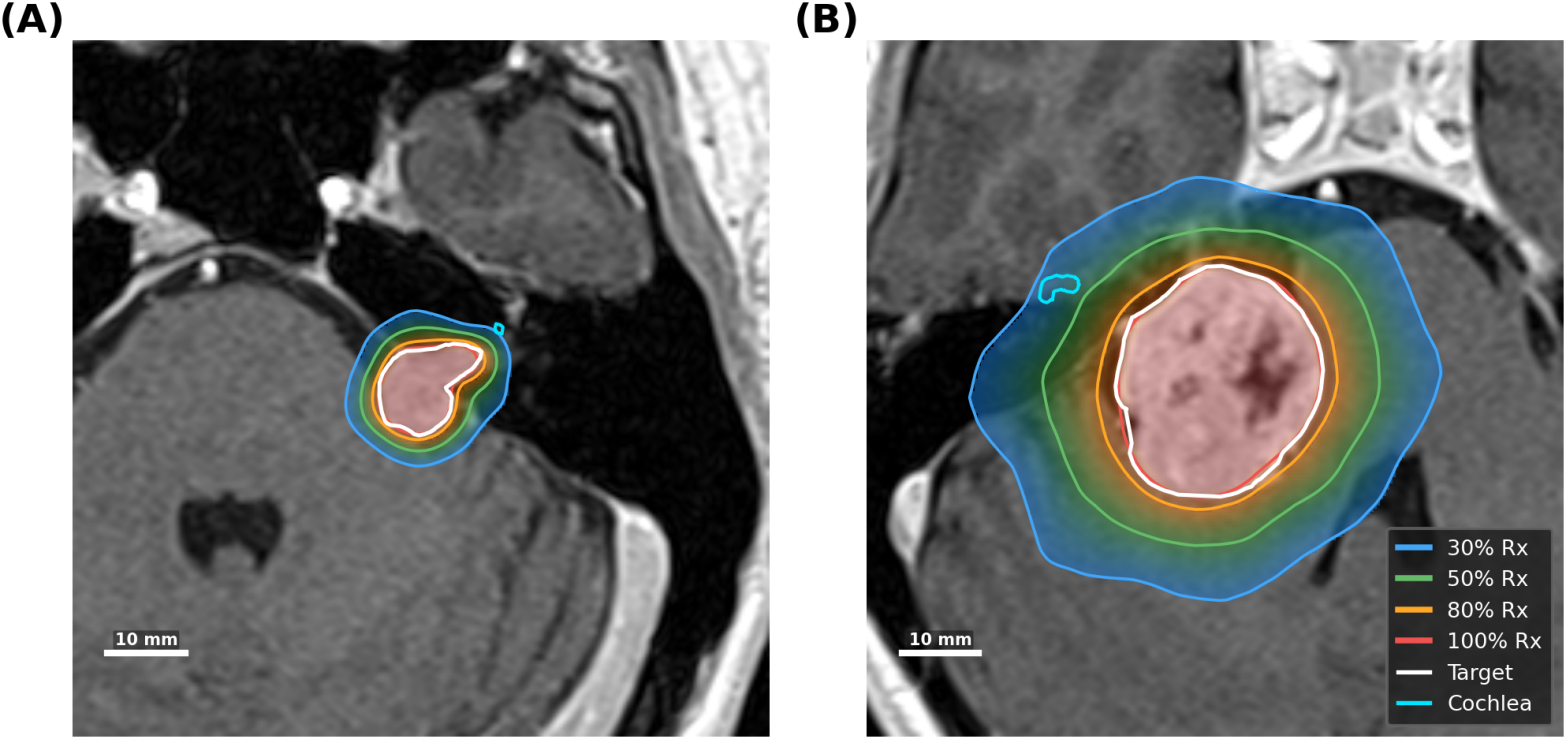
Representative axial dose distributions from VS-SEG clinical cases. **(A)** VS-SEG-023 (TV = 0.55 cc, dDGI = 0.238 mm): a small vestibular schwannoma with tightly spaced isodose lines indicating a steep dose gradient. **(B)** VS-SEG-234 (TV = 7.18 cc, dDGI = 0.562 mm): a large target with wider isodose spacing reflecting a shallower gradient. Solid lines indicate isodose levels (30%, 50%, 80%, and 100% of Rx), the target volume (white), and cochlea (cyan). T1-weighted MR images are shown as background. The scale bar represents 10 mm.

Subgroup analysis by target volume revealed systematic trends (**Table 4**): small targets (<1 cc, n=77) had the steepest gradients (dDGI = 0.229±0.045 mm) with the highest GI ( 2.856±0.240), whereas large targets ( ≥3 cc, n=22) showed shallower gradients (dDGI = 0.428±0.056 mm) and lower GI ( 2.640±0.085). **Figure 4** illustrates this volume–gradient relationship with representative axial dose distributions: VS-SEG-023 (TV = 0.55 cc, dDGI = 0.238 mm) demonstrates tightly spaced isodose lines characteristic of small-target SRS, whereas VS-SEG-234 (TV = 7.18 cc, dDGI = 0.562 mm) shows wider isodose spacing reflecting the shallower gradient associated with larger targets. Prescription dose subgroups showed no clinically significant differences: 12 Gy group (dDGI =0.358±0.111 mm) versus 13 Gy group (dDGI =0.283±0.079 mm), with the difference largely attributable to the target volume distribution.

**Table 4:** Subgroup analysis by target volume from the VS-SEG clinical dataset ((n/142)).

TV group	*n*	dDGI at Rx	GI	Paddick CI	dDGI 50% Rx
<1 cc	77	0.229 ± 0.045	2.856 ± 0.240	0.577 ± 0.094	2.403 ± 0.411
1–3 cc	43	0.344 ± 0.043	2.682 ± 0.145	0.581 ± 0.019	3.696 ± 0.479
≥3 cc	22	0.428 ± 0.056	2.640 ± 0.085	0.611 ± 0.064	4.657 ± 0.462

Values are mean ± SD.

CI, conformity index; cDGI, cumulative dose gradient index; dDGI, differential dose gradient index; GI, gradient index; Rx, prescription dose; SD, standard deviation; TV, target volume.

### Comparison with conventional indices

While CI, GI, and $R_{50}$ each evaluate dose distribution quality at one or two discrete dose levels, dDGI and cDGI provide distance-based gradient characterization at every computed dose level, yielding a complete fall-off profile (**Supplementary Table 2**).

The clinical significance of this distinction is demonstrated by the 142-case SRS analysis: dDGI at Rx was independent of Paddick CI (Pearson r=0.040, p=0.634), confirming that a plan with high target conformity does not necessarily have a steep dose gradient, and vice versa. In contrast, cDGI at 50% Rx showed a moderate negative correlation with GI ( r=−0.501), consistent with the expectation that both metrics reflect dose fall-off characteristics, albeit through different formulations (distance-based vs. volume-ratio-based).

### Uncertainty analysis

Sources of uncertainty in DGC computation were assessed using both phantom and clinical data (**Supplementary Table 3**). Grid resolution effects were quantified from TG-119 paired comparisons (1.25 vs. 2.5 mm): relative dDGI differences ranged from 0.4% (C-shape) to 10.2% (multi-target), with a mean of approximately 5%. The systematic factor between marching cubes and voxel-based surface area methods was approximately 2.5x, but this is deterministic within each method and does not affect rank-order comparisons when a consistent method is used. Volume computation showed excellent agreement (<0.6% difference between voxel-counting and mesh-based methods). T1/T2-based paired RT plans (VS-SEG, n=30) demonstrated strong computational reproducibility (dDGI: r=0.987, diff = 3.3%; cDGI: diff = 0.4%; GI: r=0.989, diff = 0.7%; CI: diff = 2.9%).

Computational reproducibility was assessed using the VS-SEG dataset, where 30 patients had matched T1- and T2-MR-based RT object sets analyzed independently. The dDGI showed excellent reproducibility (Pearson r=0.987, mean absolute difference 3.3%), as did GI ( r=0.989, difference 0.7%) and cDGI (difference 0.4%). Paddick CI showed slightly higher variability (difference 2.9%), attributable to differences in target delineation between T1 and T2 sequences.

## Discussion

This study presents an automated DGC and DVH analysis platform that addresses the two primary barriers to clinical adoption of dose gradient evaluation: computational burden and absence of clinical interpretation guidance.

### Clinical decision support

The integration of clinically informed quality grading for dDGI values provides immediate clinical context that was absent from the original DGC implementation. The four-tier grading system (Ideal/Good/Acceptable/Poor) translates the numerical dDGI into actionable plan quality assessments, enabling planners to identify suboptimal dose gradients and prioritize re-optimization efforts, complementing rather than replacing conventional DVH-based evaluation. The interpretation text is designed to provide immediate feedback to facilitate plan review, rather than acting as a strict clinical mandate.

Importantly, dDGI-based grading should be interpreted alongside conventional metrics (CI, GI, $R_{50\%}$) rather than as a replacement. The 142-case clinical SRS analysis demonstrated that dDGI is statistically independent of Paddick CI (Pearson r=0.040, p=0.634), confirming that gradient steepness and target conformity measure distinct plan quality aspects. The moderate correlation between cDGI and GI ( r=−0.501) is expected, as both capture dose fall-off characteristics, but the distance-based cDGI provides dose-level-resolved information that the single-ratio GI cannot. The web-based architecture of the platform (https://dgi.oncosoft.io) inherently supports multi-institutional data collection: any institution can upload DICOM data through a standard web browser without software installation, enabling prospective studies across diverse treatment sites and techniques.

It is worth noting that several widely used guidelines for evaluating SABR dose fall-off rely on volume-ratio metrics paired with target-size-stratified tolerance tables. The Radiation Therapy Oncology Group (RTOG) 0813 protocol for centrally located lung SBRT (18) uses $R_{50\%}$ with a PTV-stratified table in which the acceptable $R_{50\%}$ decreases from approximately 5.9 for very small PTVs (1.8–3.8 cc) to 2.9 for larger PTVs (74–163 cc). The UK SABR Consortium guidelines (v6.1, 2019) (19) similarly employ a “Modified Gradient Index” defined as Vol(50%)/ ${\text{PTV}}_{V100\%}$, with separate tolerance tables for lung and non-lung sites: for lung targets the Tolerance value decreases from 9 (Minor Deviation 9–11) for PTV<20 cc to 4.5 for PTV>90 cc, and for non-lung sites it ranges from 7.5 for PTV<20 cc to 5.5 for PTV>40 cc. Recent national SABR protocols in the UK, including the West Midlands SABR (lung) protocol v3.0 (20) and the UK SABR Consortium pelvic re-irradiation guidance (21), continue to operate within this volume- and site-stratified framework. The need for such stratification reflects the intrinsic target-volume dependence of volume-ratio metrics. Under a simple spherical approximation in which a target of radius r is surrounded by a uniform physical fall-off distance d from the prescription isodose to the 50% isodose, the corresponding volume ratio scales as ${\left(1+d/r\right)}^{3}$: an identical physical gradient (e.g., d=3 mm) yields a ratio of approximately 2.20 for a small target ( r=1 cm) but only 1.19 for a larger one ( r=5 cm). This cube-law dependence means that a physically steep fall-off can still produce a relatively large ratio when the target is small, and the sliding-scale thresholds in these guidelines may be understood, at least in part, as an adjustment for this geometric effect rather than as a prescription of stricter gradients for larger targets (10).

Because dDGI is expressed directly as the absolute physical fall-off distance in millimeters—conceptually the quantity d in the geometric relation above—it is less directly coupled to target radius and can, in principle, be applied with a single, volume- and site-agnostic reference criterion (e.g., Ideal <3 mm). This may complement the existing protocol-based $R_{50\%}$ and Modified Gradient Index criteria by providing a more directly interpretable measure of the physical steepness of dose fall-off across a wide range of target sizes and anatomical sites, without replacing the established tolerance tables that remain valuable because they are calibrated to observed clinical practice and achievable plan quality.

Whereas dDGI characterizes the gradient at a single dose interval, cDGI provides the cumulative distance from the prescription isodose surface to each lower dose level, capturing the overall shape of dose fall-off (4). Using marching-cubes-based surface area estimation, the cDGI at 50% Rx ranged from 11.7 to 15.7 mm across the TG-119 cases, reflecting plan- and geometry-dependent differences in the spatial extent of dose spread. Because the TG-119 phantoms represent conventional IMRT geometries with moderately sized targets, these values are consistent with the range expected for standard conformal plans; SRS and SBRT plans, which are optimized for very steep fall-off around small targets, would be expected to produce substantially lower cDGI at 50% Rx (on the order of 3–6 mm), consistent with the more compact dose distributions characteristic of those techniques (1, 13).

The TG-119 results illustrate that dDGI and cDGI capture complementary aspects of dose fall-off. The head-and-neck case produced the largest cDGI at 50% Rx (15.7 mm) despite having the highest dDGI at Rx (0.702 mm), indicating that a relatively gradual near-field gradient does not preclude a large total fall-off distance when the dose distribution is spatially extensive. Conversely, the prostate and multi-target cases demonstrate the opposite dissociation: the prostate plan had the steepest near-field gradient (dDGI at Rx = 0.312 mm) yet a comparable cDGI at 50% Rx (12.4 mm) to the multi-target plan (dDGI = 0.404 mm, cDGI = 12.4 mm). This contrast arises because the prostate plan surrounds a single, relatively large target whose isodose surfaces extend further in space, producing a broader overall dose cloud despite the steep initial fall-off. The multi-target plan, in contrast, delivers dose to several small, spatially separated volumes whose individual fall-off regions are compact, yielding a similar cumulative distance to the 50% Rx level even though the gradient at Rx is less steep. These examples underscore that dDGI alone does not fully predict the spatial extent of low-dose spread, and that cDGI provides actionable supplementary information for clinical plan comparison—particularly when competing plans have similar dDGI at Rx but differ in their fall-off patterns at lower dose levels.

### Computational considerations

The >99.8% reduction in computation time results from replacing the three-stage manual workflow (coordinate export, file parsing, iterative mesh computation) with a single automated pipeline. Isodose surface area is computed by default using the marching cubes algorithm, which extracts a triangulated isosurface from the binary dose mask and computes exact mesh area via scikit-image. This method adds approximately 1–2 seconds per case, keeping total computation time well below 10 seconds.

As a faster alternative, the platform also provides a voxel-based boundary counting method using six-connectivity neighbor detection. A direct comparison of the two approaches on the TG-119 datasets showed that the voxel-based method underestimates surface area by approximately 60% relative to marching cubes, primarily due to an empirical correction factor applied during boundary counting (**Supplementary Table 1**). Because surface area appears in the denominator of the dDGI formula, this underestimation inflates dDGI and cDGI values by a consistent factor of approximately 2.5× across all cases and dose levels. In contrast, voxel-counting and mesh-based volume estimates agreed to within 0.6% across all cases and dose levels at or below the prescription dose, confirming that isodose volume is insensitive to the estimation method. Although the voxel method is substantially faster (<0.3 seconds per case), the systematic surface area bias makes marching cubes the recommended default for quantitative gradient reporting.

### TG-119 validation

The TG-119 validation confirmed that the platform reproduces DVH metrics consistent with published recommendations (11). It should be noted that TG-119 validation establishes computational correctness of the platform; clinical grading thresholds require separate validation with stereotactic clinical data, which we provide in the Clinical SRS Validation section above. The dDGI at Rx varied considerably across phantom configurations, ranging from 0.312 mm (prostate) to 0.702 mm (head-and-neck) on the 1.25 mm grid. These differences reflect the distinct dose fall-off characteristics of each plan geometry: the prostate plan, with a relatively compact and convex target, produced the steepest gradient, whereas the head-and-neck plan, with a large non-convex PTV extending from the skull base to the upper neck and concave target-OAR arrangements, yielded a shallower gradient. Under the proposed quality grading, all TG-119 dDGI at Rx values fell within the Ideal category (<3 mm), which is expected for well-optimized phantom plans without the geometric complexity of clinical patient anatomy.

The magnitude of the grid effect varied markedly across cases (22, 23). The C-shape case showed near-identical dDGI values across grids (∆dDGI = 0.002 mm, 0.4%), indicating that the dose calculation grid had negligible impact for this moderately concave geometry. The prostate case showed a small difference (4.9%), with the 1.25 mm grid yielding a steeper gradient as expected. In contrast, the head-and-neck (6.1%) and multi-target (10.2%) cases exhibited the opposite trend, with the finer grid yielding higher dDGi.

For the multi-target case—comprising several small, spatially separated volumes—this likely reflects spatial aliasing effects in the inter-target dose regions, where the isodose surface geometry can shift appreciably between grid resolutions; for the head-and-neck case, the complex non-convex PTV geometry extending from skull base to upper neck may amplify sensitivity to the calculation grid. More broadly, grid resolution sets a lower bound on the spatial frequency at which the dose distribution can be resolved: plans with rapidly changing isodose geometry—whether from non-convex PTV shape, steep gradients around small volumes, or interactions between separated high-dose regions—are inherently more sensitive to grid refinement, whereas well-optimized plans with moderate gradient steepness and smooth isodose topology (as observed for the C-shape case) can yield near-identical values across grids.

Grid resolution also influenced cDGI at 50% Rx, though differences were generally smaller than for dDGI. The C-shape and head-and-neck cases showed cDGI differences below 1.5%, while the prostate and multi-target cases exhibited differences of approximately 6%. The attenuated cDGI sensitivity reflects the averaging effect of summing dDGI across many isodose levels, which smooths out local grid artifacts that dominate single-level measurements. These observations underscore the importance of reporting the dose grid resolution alongside gradient metrics when comparing treatment plans, and suggest that grid sensitivity is greatest for small targets and complex geometries with spatially separated volumes.

### Reporting recommendations

Given the grid-dependent nature of DGC metrics, we recommend the following: (1) dDGI and cDGI values must always be reported alongside the dose grid resolution; (2) for inter-institutional comparison, the finest available grid ( ≤1.5 mm for SRS/SABR, ≤2.5 mm for IMRT) should be used; (3) the observed grid sensitivity is geometry-dependent (0.4% for the C-shape to 10.2% for the multi-target case), providing a practical guide for interpreting variability. The platform displays the current dose grid resolution in the interface and exports it with CSV results. When an RT Plan file is uploaded alongside the RT Dose and RT Structure Set, the prescription dose and fractionation information are automatically extracted from DICOM metadata, eliminating the uncertainty associated with the $5/6\times D_{max}$ estimation method.

### Limitations

Several limitations should be acknowledged. First, while TG-119 phantom validation confirmed computational accuracy and the VS-SEG analysis (142 vestibular schwannoma SRS cases) demonstrated generalizability to small-volume, steep-gradient clinical geometries, broader validation across diverse treatment sites (e.g., lung SBRT, spine SRS, head-and-neck IMRT) and multiple institutions is needed to confirm that platform-computed dDGI and cDGI values correlate with treatment outcomes. Second, the four-tier quality grading offers an intuitive clinical interpretation of gradient steepness; the Ideal threshold (<3 mm) is anchored to the established SRS benchmark of Wagner et al. (8), yet broader thresholds should be refined through prospective multi-institutional validation correlating dDGI/cDGI with clinical outcomes across diverse treatment sites and techniques. Third, the platform currently evaluates a single plan at a time; in clinical practice, however, comparing gradient metrics across competing plans is essential for informed plan selection. A multi-plan comparative evaluation system is currently under development and will be supported in a future release.

## Conclusion

This study developed a web-based platform that automates DGC and DVH analysis from DICOM RT files, reducing computation time from over 2 hours to under 10 seconds. TG-119 phantom validation confirmed that all dose coverage and OAR metrics met published benchmarks, and clinical validation on 142 vestibular schwannoma SRS plans demonstrated that DGC metrics capture dose fall-off characteristics complementary to conventional conformity and gradient indices. The integrated clinical quality grading translates numerical dDGI values into actionable plan assessments, complementing conventional DVH-based evaluation. The platform is freely accessible at https://dgi.oncosoft.io.

## Data Availability

The Vestibular-Schwannoma-SEG clinical dataset used for SRS validation is publicly available from The Cancer Imaging Archive under a Creative Commons Attribution 4.0 International (CC BY 4.0) license.
