# Supplementary material for: Automated dose-gradient curve and dose-volume histogram analysis platform: development, validation, and clinical decision support with TG-119 datasets
Source: Front Oncol. 2026 May 20;16:1826856. doi: 10.3389/fonc.2026.1826856 (PMC13229744; doi:10.3389/fonc.2026.1826856)
Supplement: Supplementary file 1 [file Table1.docx]

**Supplementary materials**

**Automated Dose-Gradient Curve and Dose-Volume Histogram Analysis Platform: Development, Validation, and Clinical Decision Support**

**Table of Contents**

**Table S1.** Comparison of voxel-counting and marching cubes methods p.2

**Table S2.** Comparison of dosimetric plan quality indices p.4

**Table S3.** Uncertainty analysis for DGC metrics and conventional indices p.5

**Table S1.** Comparison of voxel-counting and marching cubes methods for isodose volume and surface area estimation across the TG-119 cases. Isodose volumes and surface areas were computed at five dose levels (25%, 50%, 75%, 100%, and 110% of the prescription dose) at both grid resolutions. Volume difference ($\Delta V$) and surface area difference ($\Delta S$) are expressed as the percentage deviation of the voxel-based value relative to the marching cubes value. Negative $\Delta S$ indicates that the voxel method underestimates surface area.

|  |  |  |  | **Volume (cm^3^)** | | | **Surface area (mm^2^)** | | |
| --- | --- | --- | --- | --- | --- | --- | --- | --- | --- |
| **Case** | **Grid (mm)** | **%Rx** | **Dose (Gy)** | **Voxel** | **MC** | $\boldsymbol{\Delta V}$ **(%)** | **Voxel** | **MC** | $\boldsymbol{\Delta S}$ **(%)** |
| Prostate | 1.25 | 25 | 17.2 | 1450.9 | 1449.6 | 0.09 | 39447 | 97318 | -59.5 |
|  | 1.25 | 50 | 34.5 | 395.0 | 394.1 | 0.23 | 14141 | 37389 | -62.2 |
|  | 1.25 | 75 | 51.8 | 171.3 | 170.8 | 0.29 | 6769 | 18061 | -62.5 |
|  | 1.25 | 100 | 69.0 | 104.3 | 103.9 | 0.37 | 4675 | 12190 | -61.7 |
|  | 1.25 | 110 | 75.9 | 83.9 | 83.6 | 0.34 | 4347 | 11450 | -62.0 |
|  | 2.5 | 25 | 17.2 | 1478.2 | 1476.9 | 0.09 | 42012 | 103111 | -59.3 |
|  | 2.5 | 50 | 34.5 | 374.5 | 373.7 | 0.21 | 13212 | 34897 | -62.1 |
|  | 2.5 | 75 | 51.8 | 172.4 | 172.0 | 0.28 | 6750 | 17838 | -62.2 |
|  | 2.5 | 100 | 69.0 | 109.0 | 108.7 | 0.33 | 4866 | 12639 | -61.5 |
|  | 2.5 | 110 | 75.9 | 88.9 | 88.6 | 0.34 | 4441 | 11695 | -62.0 |
| Head & Neck | 1.25 | 25 | 11.5 | 3477.7 | 3476.3 | 0.04 | 72647 | 164096 | -55.7 |
|  | 1.25 | 50 | 23.0 | 1779.6 | 1778.8 | 0.05 | 42272 | 101978 | -58.5 |
|  | 1.25 | 75 | 34.5 | 972.7 | 971.6 | 0.12 | 28062 | 70596 | -60.2 |
|  | 1.25 | 100 | 46.0 | 630.9 | 630.1 | 0.13 | 19941 | 49075 | -59.4 |
|  | 1.25 | 110 | 50.6 | 449.0 | 460.4 | -2.46 | 41678 | 92580 | -55.0 |
|  | 2.5 | 25 | 11.5 | 3251.4 | 3249.9 | 0.05 | 71191 | 162300 | -56.1 |
|  | 2.5 | 50 | 23.0 | 1735.9 | 1734.9 | 0.06 | 40084 | 95450 | -58.0 |
|  | 2.5 | 75 | 34.5 | 978.2 | 977.3 | 0.10 | 28041 | 69482 | -59.6 |
|  | 2.5 | 100 | 46.0 | 621.1 | 620.3 | 0.13 | 19975 | 48525 | -58.8 |
|  | 2.5 | 110 | 50.6 | 497.4 | 500.3 | -0.58 | 23403 | 52707 | -55.6 |
| C-shape | 1.25 | 25 | 11.2 | 2015.1 | 2014.1 | 0.05 | 47212 | 109665 | -56.9 |
|  | 1.25 | 50 | 22.5 | 883.3 | 882.5 | 0.09 | 27819 | 68354 | -59.3 |
|  | 1.25 | 75 | 33.8 | 378.4 | 377.6 | 0.21 | 15287 | 38411 | -60.2 |
|  | 1.25 | 100 | 45.0 | 227.2 | 226.6 | 0.28 | 10912 | 27025 | -59.6 |
|  | 1.25 | 110 | 49.5 | 183.5 | 182.9 | 0.33 | 9909 | 24572 | -59.7 |
|  | 2.5 | 25 | 11.2 | 2077.1 | 2075.8 | 0.06 | 47937 | 111770 | -57.1 |
|  | 2.5 | 50 | 22.5 | 900.0 | 899.3 | 0.09 | 27731 | 68777 | -59.7 |
|  | 2.5 | 75 | 33.8 | 388.0 | 387.2 | 0.21 | 15287 | 38611 | -60.4 |
|  | 2.5 | 100 | 45.0 | 230.9 | 230.2 | 0.27 | 10969 | 27134 | -59.6 |
|  | 2.5 | 110 | 49.5 | 189.7 | 189.1 | 0.32 | 9900 | 24739 | -60.0 |
| Multi-target | 1.25 | 25 | 11.0 | 1178.9 | 1177.4 | 0.13 | 38116 | 94208 | -59.5 |
|  | 1.25 | 50 | 22.0 | 345.9 | 345.1 | 0.23 | 13769 | 35117 | -60.8 |
|  | 1.25 | 75 | 33.0 | 118.2 | 117.7 | 0.37 | 5469 | 14081 | -61.2 |
|  | 1.25 | 100 | 44.0 | 71.2 | 70.8 | 0.51 | 3828 | 9637 | -60.3 |
|  | 1.25 | 110 | 48.4 | 57.0 | 56.7 | 0.55 | 3425 | 8457 | -59.5 |
|  | 2.5 | 25 | 11.0 | 1220.4 | 1219.1 | 0.10 | 37059 | 91225 | -59.4 |
|  | 2.5 | 50 | 22.0 | 321.2 | 320.5 | 0.21 | 12484 | 31592 | -60.5 |
|  | 2.5 | 75 | 33.0 | 115.6 | 115.2 | 0.40 | 5487 | 13854 | -60.4 |
|  | 2.5 | 100 | 44.0 | 73.8 | 73.5 | 0.48 | 4028 | 10058 | -59.9 |
|  | 2.5 | 110 | 48.4 | 59.0 | 58.7 | 0.54 | 3556 | 8607 | -58.7 |

Abbreviations: MC, marching cubes; Rx, prescription dose; %Rx, isodose level expressed as percentage of Rx; $\Delta V$, relative volume difference (voxel vs. MC); $\Delta S$, relative surface area difference (voxel vs. MC).

**Table S2.** Comparison of dosimetric plan quality indices, with DGC metrics providing distance-based gradient characterization complementary to existing volumetric indices. VS-SEG average values ($n=142$) are shown for reference.

| **Index** | **Definition** | **What it measures** | **Dose-level** | **VS-SEG** |
| --- | --- | --- | --- | --- |
| Paddick CI | $TV_{PIV}^{2} / (TV\times PIV)$ | Target conformity | Single (Rx) | $0.584 \pm0.075$ |
| GI (Paddick) | $V_{50\%Rx} / V_{Rx}$ | Volumetric fall-off | Single (50%Rx) | $2.770 \pm0.218$ |
| *R_50%_* | $V_{50\%Rx} / TV$ | Dose spillage | Single (50%Rx) | $4.256 \pm0.601$ |
| dDGI | $Shell \Delta V / \bar{S}, per\Delta d$ | Isodose surface distance | Every level | $0.294 \pm0.089$ mm |
| cDGI | $\sum\mathrm{dDGI}$ from Rx to $d$ | Cumulative distance | Every level | $3.139 \pm1.037$ mm |

Abbreviations: CI, conformity index; cDGI, cumulative dose gradient index; dDGI, differential dose gradient index; GI, gradient index; PIV, prescription isodose volume; $R_{50\%}$, ratio of 50% isodose volume to target volume; Rx, prescription dose; TV, target volume; $TV_{PIV}$, volume of target covered by the prescription isodose; $V_{50\%Rx}$, volume receiving ≥50% Rx; $V_{Rx}$, volume receiving ≥Rx; VS-SEG, Vestibular-Schwannoma-SEG dataset.

**Table S3.** Uncertainty analysis summarizing the principal sources of variability for dose gradient curve metrics and conventional indices.

| **Source** | **Method** | **Result** |
| --- | --- | --- |
| Grid resolution  (TG-119, 1.25 vs 2.5 mm) | Paired comparison, 4 cases | dDGI relative diff: 0.4% (C-shape) to 10.2% (multi-target); mean ~ 5% |
| Surface area method  (marching cubes vs voxel) | Systematic comparison | Systematic factor ~ 2.5×; deterministic within method |
| T1–T2 reproducibility  (VS-SEG, *n* = 30 paired) | Same patient, two RT plans  (T1 vs T2 MR-based) | dDGI: $r=0.987$, diff = 3.3%  cDGI: diff = 0.4%  GI: $r=0.989$, diff = 0.7%  CI: diff = 2.9% |
| Volume computation | Voxel-counting vs. mesh | Agreement within 0.6% |
| Step size | 1 Gy vs. 2 Gy steps | Negligible (normalized by $\Delta d$) |

Abbreviations: CI, conformity index; cDGI, cumulative dose gradient index; dDGI, differential dose gradient index; GI, gradient index; MR, magnetic resonance; $r$, Pearson product-moment correlation coefficient; Rx, prescription dose; TG-119, AAPM Task Group 119 test suite; VS-SEG, Vestibular-Schwannoma-SEG dataset.
